# A Propeller Perforator Flap in the Distal Lower Extremity: An Alternative to Free Flap Coverage Near the Ankle

**DOI:** 10.7759/cureus.15476

**Published:** 2021-06-06

**Authors:** William Aukerman, Daniel Urias, Bradley Winegardner, Kristopher Katira

**Affiliations:** 1 Surgery, Conemaugh Memorial Medical Center, Johnstown, USA; 2 Plastic and Reconstructive Surgery, Tulane University Health Sciences Center, Tulane Ochsner Plastic Surgery Program, New Orleans, USA; 3 General Surgery, Lake Erie College of Osteopathic Medicine, Erie, USA; 4 Plastic and Reconstructive Surgery, Ochsner Medical Center, New Orleans, USA

**Keywords:** peroneal perforator flap, locoregional flap, free tissue transfer, pedicle orientation, distal lower extremity

## Abstract

As perfusion assessment technologies and microsurgical techniques have evolved, plastic surgeons have become increasingly aggressive and creative in offering reconstructive solutions to limb salvage problems. In the distal lower extremity, pedicled perforator flap transfer has grown in popularity as compared to the historically reliable option of free tissue transfer. Pedicled perforator flaps typically avoid muscle harvest and restore the thin, supple soft tissue in the distal extremity, where there is a relative lack of redundancy of soft tissues. They also allow for a shorter operative time and recovery in otherwise complex wounds of the foot and ankle. This case report highlights the indications, nuance, and post-operative course of a patient who underwent peroneal perforator flap for coverage of a complex ankle wound in the setting of a calcaneal fracture.

## Introduction

The paradigm of lower-extremity soft tissue coverage has drastically evolved since microsurgical techniques were first introduced into clinical practice in the 1970s and 1980s [1]. Traditionally, lower-extremity reconstructive options have been divided into three zones, with free tissue transfer being taught as the most reliable option for coverage of wounds in the distal third of the lower extremity [2]. Perforator flaps are defined as a locoregional flap containing skin, subcutaneous tissue, muscle, and/or fascia, which are isolated on the dominant perforating vessel of a perforasome [3,4]. In comparison to traditional musculocutaneous flaps, perforator flaps provide the advantages of greater degrees of freedom in tissue motion around the flap blood supply, preservation of the underlying vascular pedicle continuity, and avoidance of muscle harvest [5]. 

These same qualities make perforator flaps an appealing alternative to free tissue transfer in cases of extremity wound coverage [6]. Following broad plastic surgery principles, perforator flap transfer requires a tension-free inset, healthy flap perfusion, and good venous outflow. For surgeons comfortable with perforator dissection techniques, propeller fasciocutaneous flaps provide the opportunity for comparable soft tissue coverage, shorter operative times, and potentially a less restrictive recovery compared to free tissue transfer.

## Case presentation

**Figure 1:**
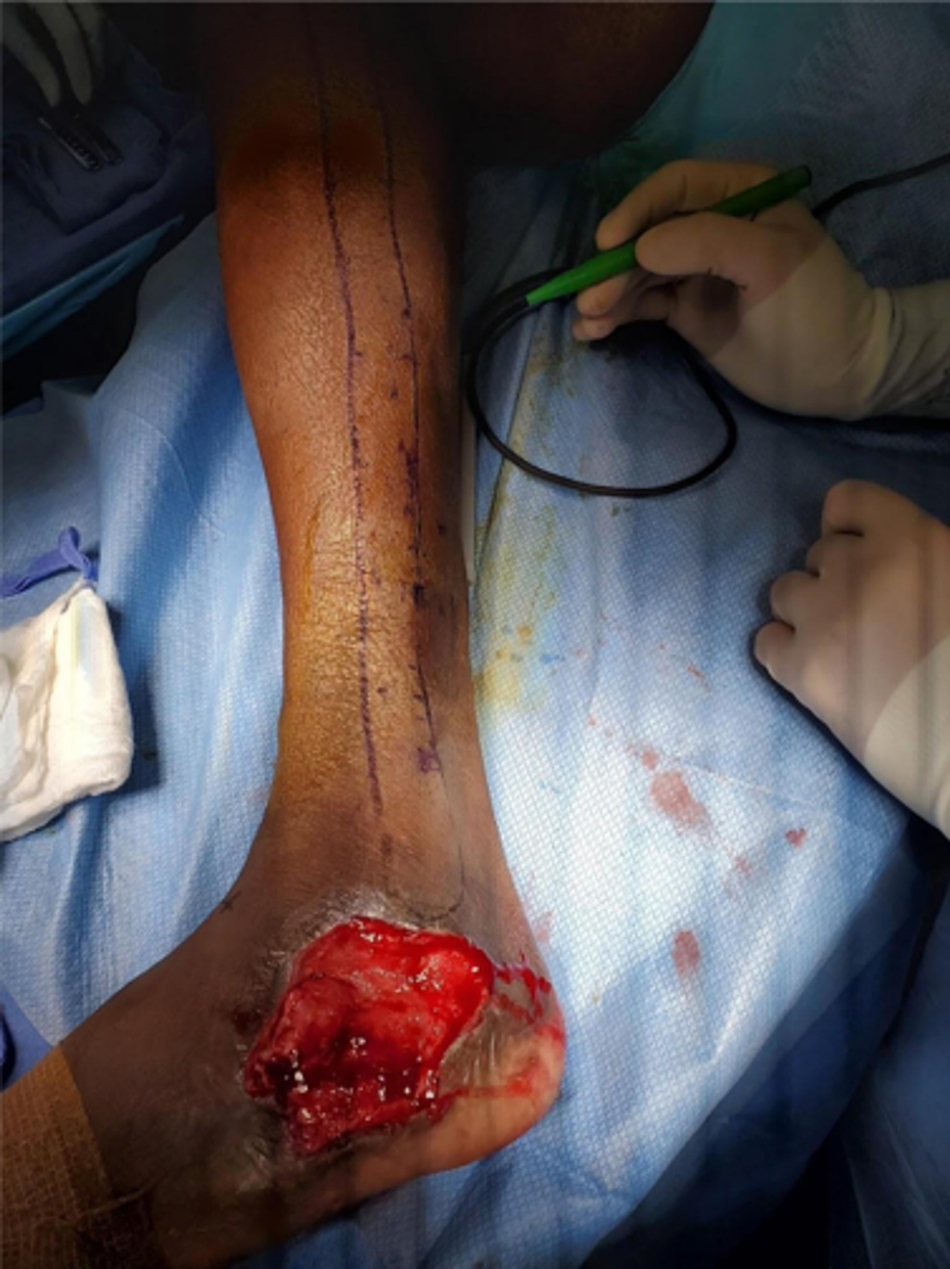
Lateral ankle and foot wound with arthrotomy

This patient initially underwent closed management of a non-displaced calcaneal fracture. After a brief period of immobilization, he developed skin breakdown and infection, necessitating operative debridement and arthrotomy with ankle irrigation. After serial washouts, plastic surgery was consulted for soft tissue coverage (Figure 1).

**Figure 2:**
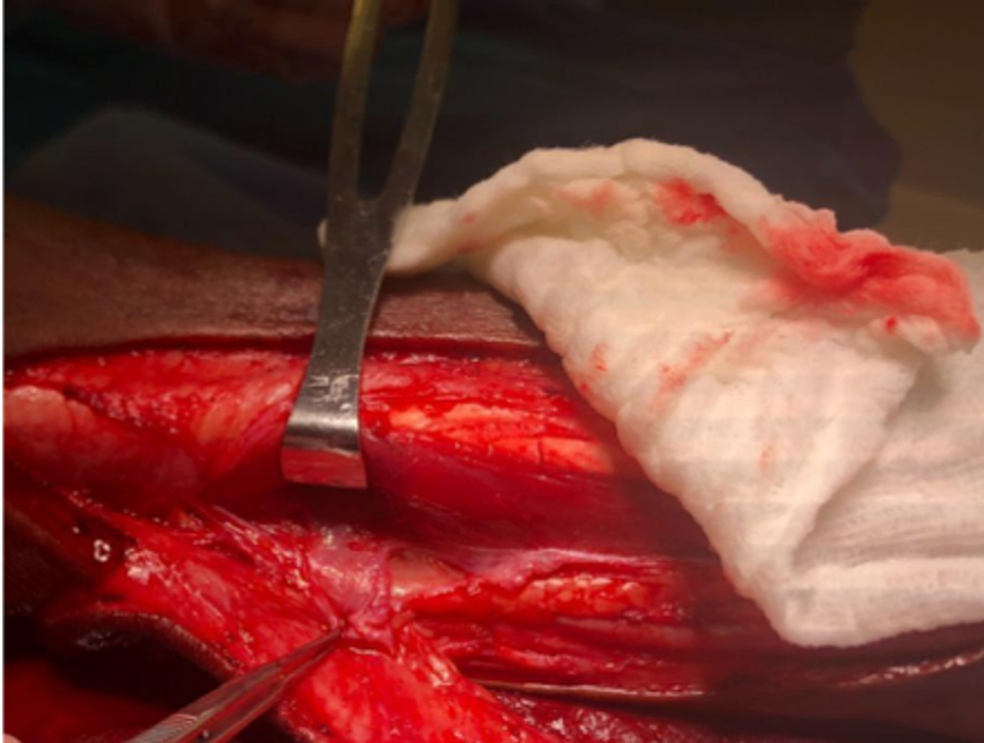
Peroneal perforator dissected eccentrically relative to skin flap

The defect and tissue laxity were analyzed. Although a posterior tibial perforator flap was considered, it was unlikely to reach in order to cover the defect. Doppler ultrasound was used to locate the perforators. The perforator flap was then marked over the perforators. Anterior incision was made, and peroneus muscles reflected anteriorly. Posterior incision following with perforators dissected out. Perfusion was then assessed. The flap was then transposed in to the defect. This peroneal perforator flap was designed to cover the arthrotomy and the remaining wound was skin grafted, including the proximal flap donor site (Figure 2).

**Figure 3:**
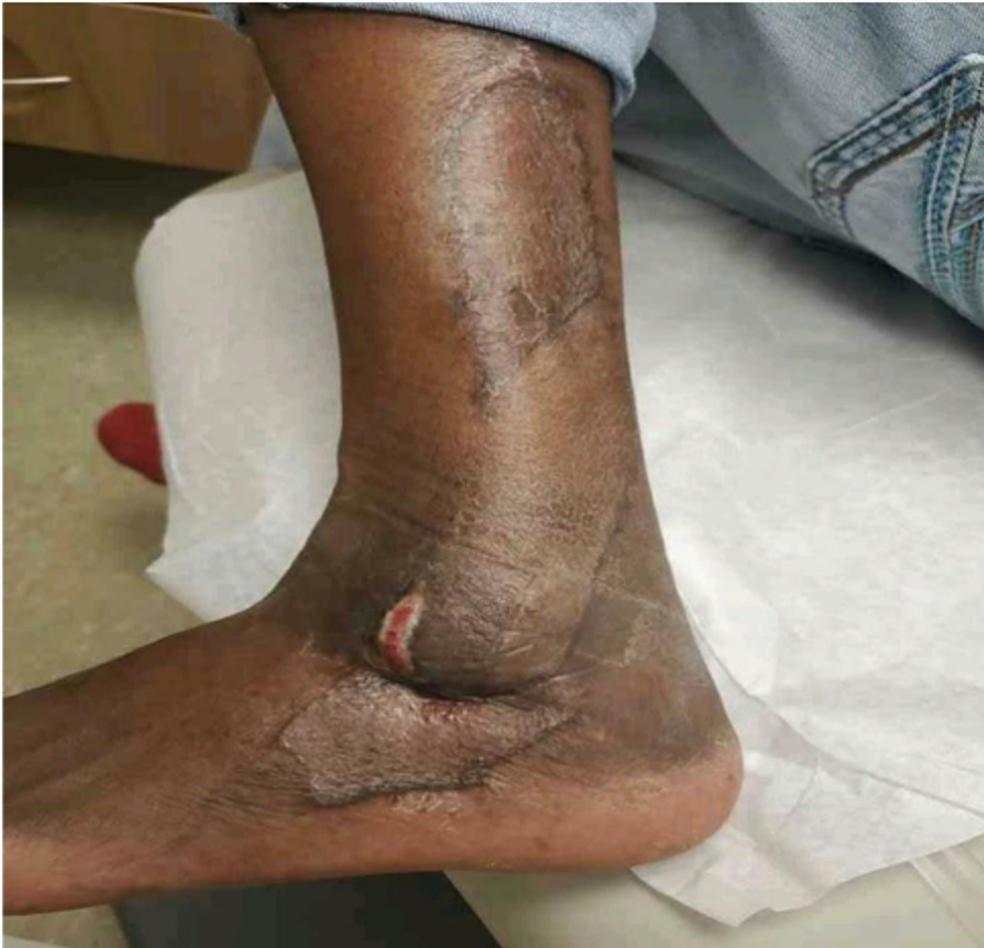
Partial-thickness loss of tip of perforator flap, managed with dressing changes

The patient was discharged home on postoperative day 5 after skin graft bolsters were removed with no limitations. Partial-thickness skin necrosis at the tip of the flap was managed with dressing changes (Figure 3).

**Figure 4:**
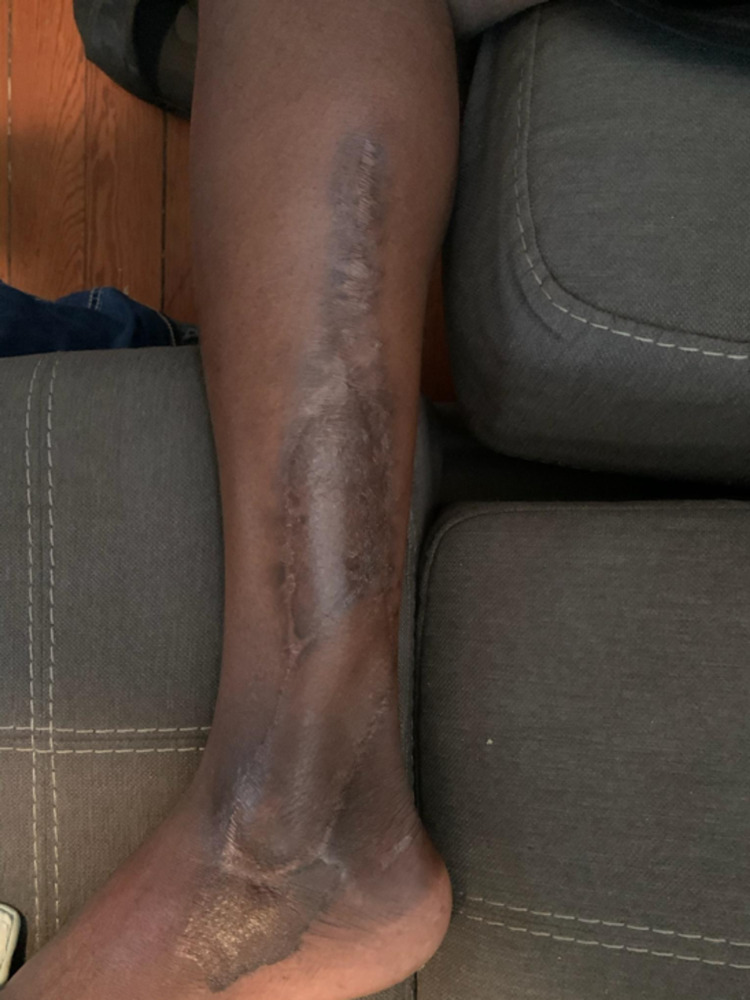
Follow-up picture showing complete healing and coverage of soft tissue defect

There was no formal dangling protocol postoperatively. The patient received culture-directed intravenous antibiotics postoperatively and began weight-bearing on schedule at the discretion of orthopedics. He did not require further orthopedic interventions (Figure 4).

## Discussion

Literature pertaining to limb reconstruction has largely focused on flap surface area, complication rates, and the timing of soft tissue coverage [7]. Studies pertaining to the efficacy, technical nuance, and indications of perforator flaps are not sparse [8]. However, perforator flap surgery is not done at all centers [9]. 

In this instance, a perforator flap was chosen over a free flap in order to avoid a longer operative time, postoperative dangling restrictions, donor-site morbidity, and recipient vessel morbidity. Most free flap surgeons employ a dangling protocol or extended periods where the limb cannot be left dependent postoperatively [10]. This patient was left non-weight-bearing at the discretion of his orthopedic providers and was advised to elevate the affected limb when non-transferring. In most centers, free tissue transfer also requires careful operative planning, specialized operating room staff and instrumentation, and microsurgical staff support that may not be possible without advanced planning or in community hospital settings [9,11]. Other than fine instrumentation, this surgery can be done at most centers, as long as the indications for the procedure are understood.

A distal third of lower-extremity defect can be a challenging case for a plastic surgeon without microvascular surgical experience. Perforator flaps may not be appropriate for some defects, as flaps are smaller and dependent on perforator topography. Some authors have argued that perforator flaps, in general, should be reserved for small- to medium-sized defects in the extremities defined as 14.8-1240.0 cm^2^ [12]. From this standpoint, perforator flaps can be raised as surgeons approach recipient vessels, under the guidance of CT, ultrasound, or indocyanine-green angiography [13].

As was noted in this case, venous congestion and partial flap necrosis are not uncommon in perforator flaps. According to a study by Koh et al., in which retrospective data, involving a small cohort of 433 patients, were analyzed, one third of the patients developed venous congestion, but no flap losses were observed [9]. 

Perhaps not coincidentally, partial flap necrosis rates secondary to venous congestion have been reported to be as high as 37.5% in some series [14]. In a European meta-analysis by Bekara et al. investigating failure rates of perforator propeller flaps in the distal third of the lower leg when compared to free flaps, partial flap necrosis was noted to be significantly higher in pedicled-propeller flaps (6.88% vs. 2.70%, P = 0.001) but soft tissue coverage was not impacted (2.99% [95% confidence interval {CI}: 3.68-6.81] vs. 5.24% [95% CI: 0.38-5.60], P = 0.016) [15]. It is possible to draw a connection between the high rates of venous congestion and partial flap necrosis in these cases. Tissue loss may be related to the eccentricity of perforators in propeller designs, venous congestion associated with wide rotation arcs around the dissected perforator, incomplete dissection of the perforator at the point of inset, or unacceptable tension at the time of inset [16]. An alternative explanation is that propeller designs themselves do not allow for tension offloading at the points of inset compared to other rotation or transposition flap patterns [12]. Experienced perforator flap surgeons have suggested that acute angle rotations are preferable over more obtuse angles [17]. Compared to free flaps, these minor complications may be more common [18]. However, delayed healing from partial flap necrosis can cause significant patient distress and utilization of healthcare resources.

One meta-analysis by Bekara et al. consisting of 40 articles, comprising 428 perforator propeller flaps, comparing outcomes between free flaps and perforator flaps, concluded that these procedures were comparably difficult and did not have significant differences in primary outcome and complication rates (overall failure rate reported as 3.9% [95% CI: 2.6-5.3] for free flaps and 2.77% [95% CI: 0.0-5.6] for pedicled-propeller flaps, P = 0.36) [8]. 

There are many options when planning a soft tissue coverage repair. The lower extremity, especially distal, has previously presented many variables hindering the ability to make up for tissue loss. The emergence of the use of perforator flaps for the distal lower extremity has recently erupted within the literature. When choosing these flaps, AlMugaren et al. suggest choosing a flap that is “technically simple, single-staged, replaces like with like, minimizes donor-site morbidity, and results in a functional, esthetic outcome” [13]. In our case, other lower-extremity flap options included posterior tibial, anterior tibial, and reverse sural flaps. The decision was made utilizing one of the three major vessels with closest proximity to the defect, allowing for a shorter arc, achieving a reliable reconstruction with like-to-like principles, decreasing dead space, utilizing more lax tissues, and dissecting the flap in a way to allow for adequate venous drainage [13,19].

Perforator-based flap repair has been increasingly described for soft tissue defects and with any advancement comes the question. A common principle found when managing similar injuries as in this case is providing sufficient amount of tissue, thus ensuring adequate arterial inflow and venous outflow. In attempts to decrease vessel sacrifice and thereby reduce flow rate complications, two-dimensional contrast radiography has previously been the standard [18]. Saint-Cyr et al. argue that three-dimensional CT, showing sagittal, coronal, and axial views, better delineates the real-time evaluation of flap vascularity [18]. These scans would allow for the visualization of areas at risk for partial-thickness necrosis. In areas in which flow is reduced, alternative coverage options may allow for better surgical outcomes. Using the vessel scans preoperatively would allow surgeons to see the perforator filling patterns, adjacent communicating vascular branches, and modeling of venous drainage, thus reducing common complications associated with perforator flap repairs [18]. 

## Conclusions

As it pertains to successful flap transfer and healing of small-to-moderate defects in the extremity, the reconstructive surgery literature suggests that perforator flap repair and free tissue transfer may be comparable. The added operative time, specialized staffing, and instrumentation needed for free tissue transfer procedures may warrant consideration of new strategies for wound coverage, such as perforator flaps, among reconstructive surgeons. The esthetic and functional consequences of perforator flap designs should be recognized when planning flaps, as potential donor-site morbidity is commonly seen with a propeller perforator flap. In this case, a peroneal perforator flap allowed the patient to heal without major vessel sacrifice, dangling restrictions, and prolonged inpatient stay or intensive nursing care. Perforator flaps should be recognized as a viable wound coverage strategy to address complex, mild- to moderate-sized extremity wounds.
